# Supplementary material for: Suppressing phagocyte activation by overexpressing the phosphatidylserine lipase ABHD12 preserves sarmopathic nerves
Source: iScience. 2025 May 9;28(6):112626. doi: 10.1016/j.isci.2025.112626 (PMC12150055; doi:10.1016/j.isci.2025.112626)
Supplement: Document S1. Figures S1 and S2 [file mmc1.pdf]

iScience, Volume *28*

Supplemental information

Suppressing phagocyte activation

by overexpressing the phosphatidylserine lipase

ABHD12 preserves sarmopathic nerves

Caitlin B. Dingwall, Yo Sasaki, Amy Strickland, Tong Wu, Daniel W. Summers, A. Joseph Bloom, Aaron DiAntonio, and Jeffrey Milbrandt

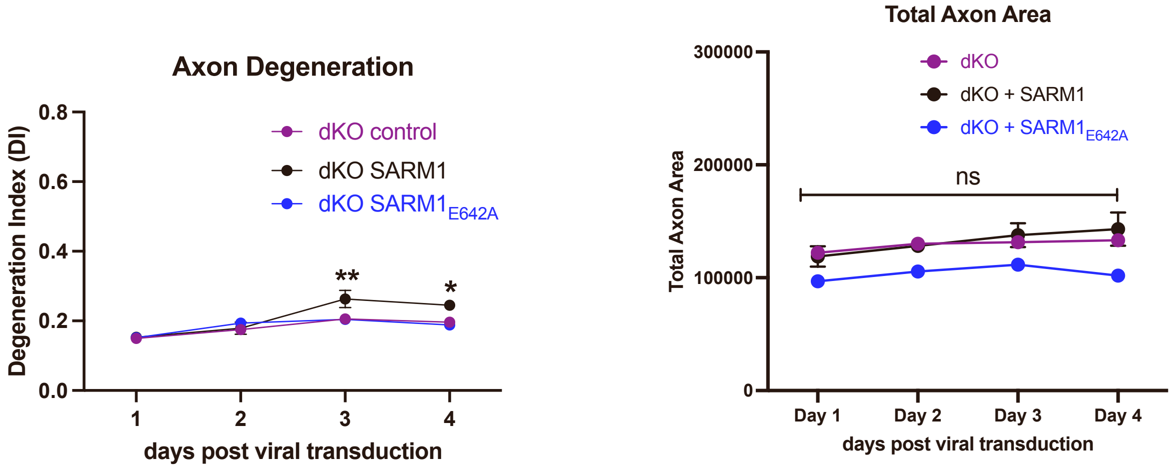

Supplemental Figure S1. SARM1 re-expression does not induce axon degeneration or reduce total axon area in dKO axons in vitro. Quantification of axon degeneration after transduction of dKO DRG neurons with control, SARM1, and SARM1$_{E642A}$ constructs using the degeneration index (DI) (n=3). All data are presented as mean ± SEM. Statistical significance determined by 2-way ANOVA. ns: not significant, *p<0.05, **p<0.01.

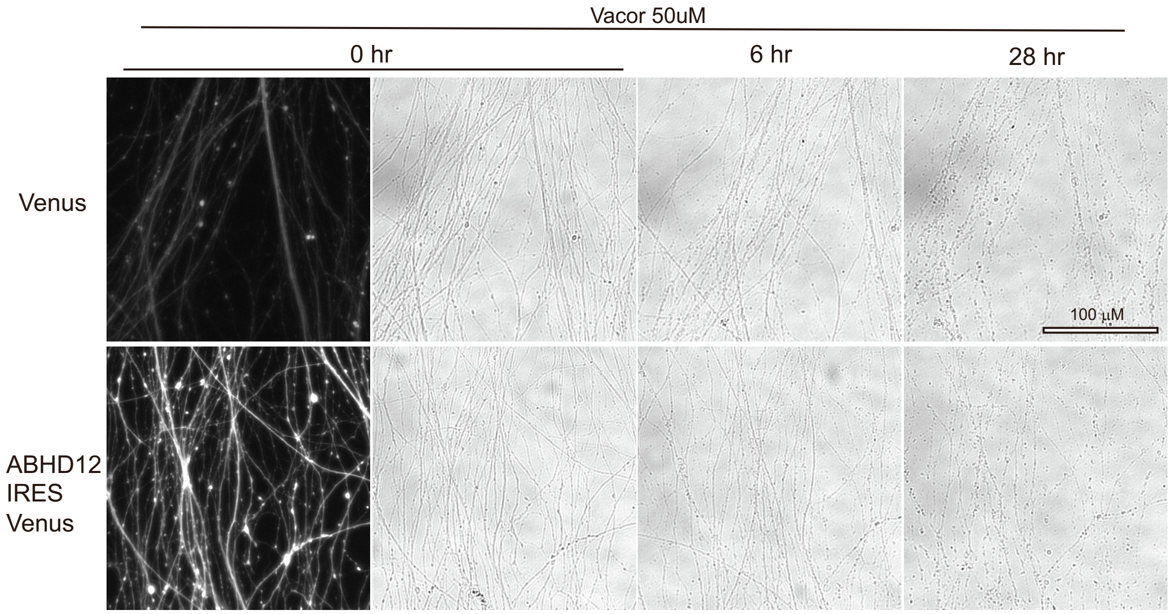

Supplemental Figure S2. ABHD12 overexpression does not protect axons from SARM1 activation in vitro. Axons from WT DRG neurons transfected with Venus (control) and ABHD12 at time 0, 6, and 28 hours after inducing SARM1 activation with 50µM Vacor.
